# The association between lipid-related obesity indicators and severe headache or migraine: a nationwide cross sectional study from NHANES 1999 to 2004

**DOI:** 10.1186/s12944-025-02432-w

**Published:** 2025-01-11

**Authors:** Xu Sun, Jimei Song, Rixun Yan, Jianwei Diao, Yibo Liu, Zhangzhi Zhu, Weichi Lu

**Affiliations:** 1 Shandong University of Traditional Chinese Medicine, Jinan, China; 2 Guangzhou University of Chinese Medicine, Guangzhou, China; 3 Department of Endocrinology, The First Affiliated Hospital of Guangzhou University of Chinese Medicine, Guangzhou, China; 4 Guangdong Clinical Research Academy of Chinese Medicine, Guangzhou, China

**Keywords:** Obesity, Lipid-related obesity indicators, Severe headache or migraine, Cross-sectional study, NHANES

## Abstract

**Background:**

The connection between lipid-related obesity indices and severe headache or migraine in young and middle-aged people aged 20–60 remains ambiguous, and there are gaps in the discriminative ability of different indicators for severe headaches or migraines. Consequently, we set out to look into this association utilizing National Health and Nutrition Examination Survey (NHANES) data from 1999 to 2004.

**Methods:**

After the values of waist-to-height ratio (WHtR), body-mass index (BMI), body roundness index (BRI), visceral adiposity index (VAI), lipid accumulation product (LAP), triglyceride glucose index (TyG), cardiac metabolism index (CMI), waist triglyceride Index (WTI), conicity index (CI) and weight-adjusted waist index (WWI) were estimated, with minimal sufficient adjustment for confounders determined by directed acyclic graph (DAG), weighted univariable and multivariable logistic regression analyses were carried out to ascertain the relationship between them and migraine. Stratified analysis and cross-effect analysis were implemented to examine the variability of intergroup correlations. Restricted cubic splines (RCS) and receiver operating characteristic (ROC) were then employed to examine nonliner relationships and its discriminatory ability for severe headache or migraine, respectively.

**Results:**

3354 United States adults were involved in our study, of whom 839 (25.01%) had severe headache or migraine. After adjusting for relevant covariables, WHtR, BRI, BMI, LAP, WTI and VAI were all associated with migraine and WHtR (OR = 6.38, 95% CI: 2.25,18.09, *P* < 0.01) showed the best predictive ability. Additionally, WHtR, BMI, and BRI demonstrated linear dose-response relationships with the prevalence of migraine (all *P*_overall_ < 0.05, *P*_non−linearity_ > 0.05).

**Conclusions:**

Among those ten lipid-related obesity indicators evaluated in the study, WHtR, BMI and BRI demonstrated linear positive dose-response relationships with the prevalence of migraine in young and middle-aged individuals within the United States and WHtR showed the best predictive ability. Our study can provide important insight into epidemiological research and comprehensive management of obese patients with migraine.

**Supplementary Information:**

The online version contains supplementary material available at 10.1186/s12944-025-02432-w.

## Introduction

Migraine and obesity are two widespread prevalence disorders which imposed substantial burdens on socioeconomic costs and patient morbidity. Migraine is a neurovascular condition that often results in disability and lowers quality-of-life [1]. Epidemiological researches have indicated that the epidemic of migraine varies between 12 and 18%, which is the primary factors contributing to disability among individuals under 50 years old, therein women experience two to three times incidence than men [2–4]. Over a third of individuals worldwide are overweight or obese [5]. Obesity commonly coexists with several clinical conditions, such as low glucose tolerance, and metabolic diseases, mental health issues, and pain syndromes [6, 7]. Identifying factors to predict the risk of migraine is vital for promoting young and middle-aged people early prevention of migraine.

Some evidence reported that the metabolic syndrome (Mets), including obesity, and dyslipidemia, are shared by migraineurs [8, 9]. Recently, obesity-migraine correlation has been comprehensively investigated. In general, obesity is a mutable hazard element that influences migraine and obesity level is correlated with the frequency, severity, duration of migraine attacks, and degree of disability [10]. The mechanism underlying the obesity-migraine nexus is probably to be intricate and multidimensional, involving disturbances in lipid metabolism, heightened secretion of pro-inflammatory compounds, nerve inflammation, and the modulation of hypothalamic neuropeptide activity [11, 12].

Lipid-related obesity indicators are low-cost, non-invasive and simple to calculate by integrating various anthropometric indices and related laboratory indicators, which have been widely utilized in the quantification of obesity and the evaluation of related disease predisposing factors. The body-mass index (BMI) is broadly applied in detecting total body obesity due to its simplicity in height and weight calculations. Emerging evidence suggests that BMI might be a credible surrogate marker for detecting severe headache or migraine [13]. Furthermore, adipose tissue distribution in obese patients is critical for migraine occurrence and development, the effectiveness of visceral fat loss in migraine improvement has gradually been confirmed. Nevertheless, previous studies primarily focused on the impact of general obesity on migraine attack characteristics, with little emphasis on central obesity and adipose tissue distribution. In addition, relying solely on one single anthropometric measure makes it challenging to identify migraine patients in a timely and precise approach. To understand the connection between these two disorders, it is imperative to investigate superior anthropometric indicators of central obesity and visceral fat to comprehensively evaluate the discriminative power of different indicators for migraine and thus promote migraine self-management. In addition, anthropometric measures have been demonstrated to lack specificity in assessing obesity-related risk in older adults due to susceptibility to metabolic disorders and abnormal fat distribution.

BMI is widely used to quantify the degree of total body obesity (TBO), and WHtR is considered as a surrogate index for assessing abdominal obesity (Abd-O). New anthropometric indices such as triglycerid-glucose (TyG) index, lipid accumulation (LAP) body roundness index (BRI), visceral adiposity index (VAI), cardiac metabolism index (CMI), waist triglyceride index (WTI), weight-adjusted waist index (WWI) and cone index (CI) are explored and proven to be more correlated with metabolic abnormality than traditional anthropometric indices, and have been used as proxies for central obesity [14–20]. Herein, this study intended to comprehensively evaluate the diagnosis significance of those ten lipid-related obesity indicators for severe headache or migraine in young and middle-aged Americans, and compared the diagnostic utility of these measures.

## Methods

### Data origin and study participants

The United States conducts a nationwide representative cross-sectional study on citizens who are not institutionalized called the National Health and Nutrition Examination study (NHANES), which employs a multistage, stratified, standardized research design. The survey covers in-home interviews, laboratory testing and health checks, including signed informed permission from each participant. On its website (https://www.cdc.gov/nchs/nhanes/), methodological informations are provided.

**Fig. 1:**
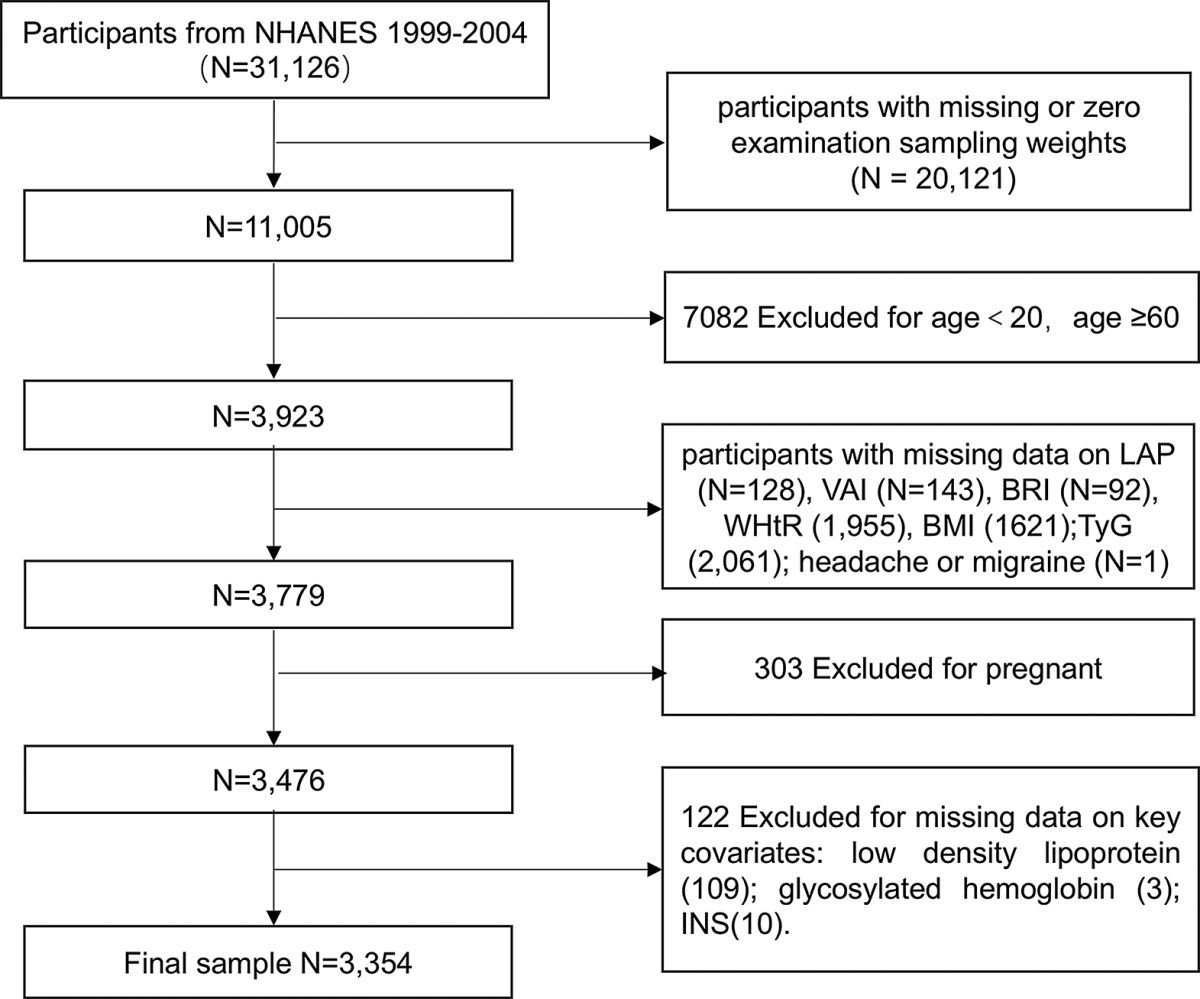
A flowchart showing the selection of study participants

This analysis focused on ten lipid-related obesity indicators on the subject of severe headache or migraine. The statistics employed in the analysis was drived from the NHANES database with 3 stages (1999–2004, including 31,126 participants). The research precluded individuals absenting or zero equalized sampling fasting weights data (*n* = 20,121). Additionally, The study removed participants aged 20 years or younger and those aged equal or older than 60 years (*n* = 7,082). Furthermore, The analysis excluded individuals with missing data on LAP (*n* = 128), VAI (*n* = 143), BRI (*n* = 92), WHtR (*n* = 92), BMI (*n* = 50), headache or migraine (*n* = 1), pregnant (*n* = 303), and key covariables (*n* = 122), specifically, low density lipoprotein (LDL) (*n* = 109), glycated hemoglobin A1c (HbAlc) (*n* = 3); fasting insulin (FSI) (*n* = 10). Ultimately, 3,354 participants (weighted *n* = 142,221,247) were incorporated into the final analytic cohort. The study procedure is depicted in Fig. 1.

### Assessment of exposure variable and outcome variables

Referring to the previous literature and recognized indicators for the prevalence of obesity, ten lipid-related obesity indicators including WHtR, BMI, VAI, BRI, LAP, TyG, CMI, WTI, CI, and WWI were included as exposure variables in our study. The quartiles of those variables were used to classify the individuals into four categories (Q1, Q2, Q3, Q4), which the the first quartile (Q1) group serving as the control group. WHtR, BMI, VAI, BRI, LAP, TyG, CMI, WTI, CI and WWI were mathematically calculated using the following formula: (1) WHtR = WC (cm)/Height (cm); (2) BMI = Weight (kg)/Height^2^(m^2^); (3) Males: VAI = WC/[39.68 + (1.88 × BMI)] × (TG /1.03) × (1.31 / HDL); Females: VAI = WC/[36.58 + (1.89 × BMI)] × (TG /0.81) × (1.52/ HDL); (4) $\:\text{B}\text{R}\text{I}=364.2-365.5\sqrt{1-\left(\frac{WC\div{\left(2\pi\:\right)}^{2}}{{\left(0.5\times\:Height\right)}^{2}}\right)}$; (5) Males: LAP = [WC (cm) – 65] × TG (mmol/L); Females: LAP = [WC (cm) – 58] × TG (mmol/L); (6) TyG index = Ln [(TG (mg/dl) × glucose (mg/dl)/2)]; (7) CMI = TG (mmol/L)/HDL (mmol/L) × [WC (cm)/HT (cm)]; (8) WTI = Ln [TG(mg/dL) × WC(cm)/2]; (9):

$$\:\text{C}\text{I}=\frac{W{C}_{\left(cm\right)}}{10.9\times\:\sqrt{\frac{Weight_{\left(kg\right)}}{Height_{\left(cm\right)}}}};\:\left(10\right)\:\:\text{W}\text{W}\text{I}\left(\text{c}\text{m}/\sqrt{\text{k}\text{g}}\right)=\text{W}\text{C}/\sqrt{\text{W}\text{e}\text{i}\text{g}\text{h}\text{t}}$$

The patients underwent laboratory testing and physical examinations at the mobile examination center (MEC) and laboratory, respectively, to obtain the data required for the ten index computations, including height (HT), body weight (WT), waist circumference (WC), high density lipoprotein cholesterol (HDL), Triglycerides (TG), fasting blood glucose (FBG).

The outcome variables of this study were the prevalence of severe headache or migraine. Severe headache or migraine were assessed by those participants who responded affirmatively to the miscellaneous pain segment of the questionnaire: “ Have you had a severe headache or migraine in the past three months? ”, which were assessed according to the the international classification of headache disorders, 3rd edition (ICHD-3) and other pertinent studies [21]. Although the NHANES did not provide more information on headache-related questions, the American migraine prevalence and prevention (AMPP) study indicated that among the participating 17.4% of patients who self-reported “severe headache,” 11.8% met the international classification of headache disorders, 2rd edition (ICHD-II), 4.6% met the criteria for “possible migraine” [22]. Therefore, it is reasonable to classify patients who report severe headache or migraine as likely to have migraine.

### Covariables

**Fig. 2:**
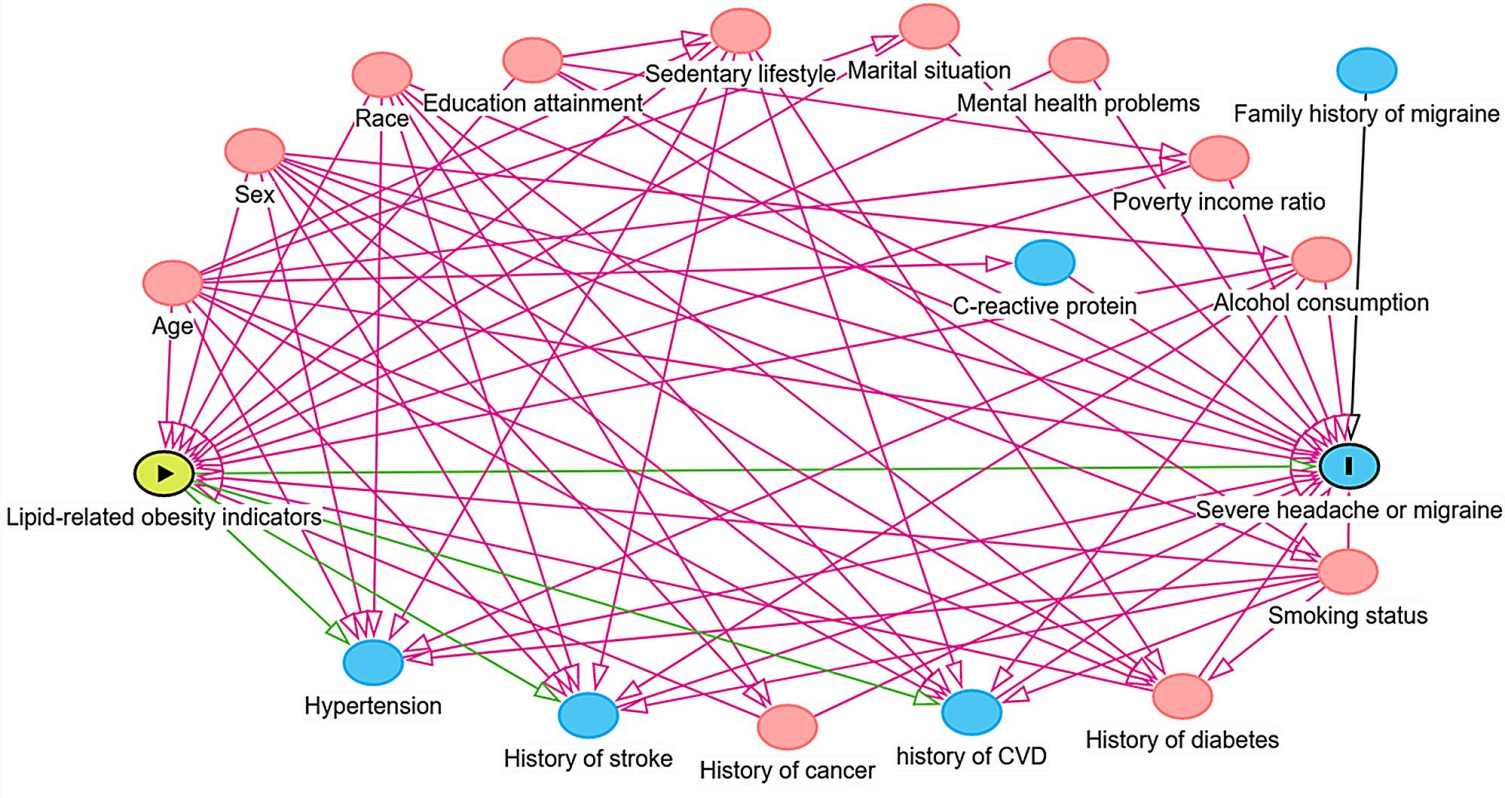
Directed acyclic graph (DAG) of the association between **lipid-related obesity indicators** with **severe headache or migraine**. Note: The variable in green and with the “►” symbol inside the rectangle was the exposure variable; the in blue and with the letter “I” inside the rectangle was the outcome variables; variables in blue are the antecedents of the outcome variable; and those in red are antecedents of the outcome and exposure variable

Based on clinical plausibility and previously published literature, the DAG was constructed using the DAGitty software (version 3.1, http://www.dagitty.net/dags.html) to identify minimally sufficient adjustment sets of covariates (MSAs) that would estimating the unconfounded effect of lipid-related obesity indices on severe headache or migraine (Fig. 2.) [23]. MSAs include demographic characteristics (age, gender, and race), educational background, marital status, income status. Additionally, some recognized clinical variables such as alcohol consumption, smoking status, sedentary behavior, history of type 2 diabetes (T2DM), mental health problems and cancer.

In this research, gender was divided as males and females. Races were designated as five groups including non-Hispanic White, Mexican American, non-Hispanic Black, other Hispanic, and other races. There were three subdivided educational attainment categories: less than high school, high school or equivalent, and college or above. Marital statuses were classified into widowed, married, separated, divorced, living with their partners, never married, and others. Furthermore, poverty income ratio (PIR) was divided into three categories: < 1.3, 1.3–1.8, and > 1.8. Alcohol consumption were categorized as never, moderate, and heavy. Smoking status was grouped into low level, moderate level, and high level [24]. If the participant’s level of physical activity during the preceding month was neither vigorous nor moderate, it was confirmed that they were engaging in sedentary behavior. Furthermore, FPG > 7.0 mmol/L, HbA1c ≥ 6.5%, or past medical history of diabetes were the criteria used to identify T2DM. Cancer was assessed by the participants who responded affirmatively to the question: “ Ever told you had cancer or malignancy?”. Mental health problems was defined by the participants had seen or talked to a mental health professional during the past 12 months.

### Statistical analysis

Based on the recommendations of the NHANES analytical guidelines, the appropriate weighting, clustering, and stratification methodology was incorporated to ensure nationally representative results. Weighted mean ± standard error (SE), weighted frequency (%) and 95% confidence interval (95% CI) were used for continuous and categorical variables, respectively. Weighted chi-square tests were performed for within-group and between-group comparisons. A series of weighted univariable and multivariable logistic regression analyses were performed to assess the relationships between lipid-related obesity markers and migraine, progressively adjusting for possibile confounding (models 1 to 3). No consideration of confounding factors in Model 1. Model 2 excluded the effects of age, race, and gender. Furthermore, model 3 excluded the effects of MSAs. The pattern of categorical variables was used to fill in missing values.

Subsequently, subgroup stratified analyses and interaction analyses were implemented within the final model to identify whether the causality varied depending on potential moderating effects of covariables. This study results are deemed significant if the interaction *P*-value < 0.05. In addition, adjusted restricted cubic splines (RCS) regression analysis was performed to account for the potential dose–response relationship between lipid-related obesity indicators with severe headache or migraine. The likelihood ratio test was utilized to assess non-linearity, and a nonlinear dose-response relationship was suggested when *P*_overall_ < 0.05 and *P*_non – linearity_ < 0.05. Furthermore, receiver operating characteristic (ROC) curves were used for diagnostic value analysis, and the area under the curve (AUC) was computed. All Statistical analysis assessments were implemented using R (version 4.2.2, http://www.R-project.org) and EmpowerStats (version 3.4.3, www.empowerstats. com), with a two-sided *P*-value threshold of less than 0.05 deemed statistically significant.

## Results

### General attributes of the study sample

The demographic characteristics and laboratory data of the included individuals with or without migraine are summarized in Table 1. We grouped these participants based on whether they had migraine and then compared their baseline conditions.

**Table 1:** General characteristics of the study participants (*n* = 3,354) in NHANES 1999–2004

Characters	Total (*n* = 3,354)	Severe headache or migraine (*n* = 839)	Non-severe headache or migraine (*n* = 2,515)	*P* value
Sex				< 0.01
Man	49.54(47.56–51.52)	34.17(30.53–38.01)	54.60(52.31–56.87)	
Female	50.46(48.48–52.44)	65.83(61.99–69.47)	45.40(43.13–47.69)	
Age	39.12 ± 0.22	39.00 ± 0.41	39.16 ± 0.26	0.71
< 40 years	51.16(49.18–53.14)	51.72(47.74–55.68)	50.97(48.69–53.25)	
40–59 years	48.84(46.86–50.82)	48.28(44.32–52.26)	49.03(46.75–51.31)	
Race/ethnicity				0.55
Mexican American	8.23(7.59–8.92)	9.03(7.70-10.55)	7.97(7.25–8.75)	
Other Hispanic	5.63(4.73–6.68)	5.92(4.12–8.45)	5.53(4.54–6.72)	
Non-Hispanic White	69.76(68.12–71.36)	67.65(64.16–70.96)	70.45(68.58–72.26)	
Non-Hispanic Black	11.46(10.59–12.39)	11.72(10.00-13.70)	11.37(10.38–12.44)	
Other races	4.93(4.11–5.89)	5.68(4.03–7.95)	4.68(3.78–5.78)	
Education				< 0.01
Less than high school	17.99(16.65–19.43)	23.91(20.88–27.22)	16.05(14.59–17.62)	
High school or equivalent	25.10(23.40-26.87)	26.43(23.08–30.07)	24.66(22.71–26.71)	
College or above	56.73(54.77–58.66)	49.49(45.53–53.46)	59.11(56.87–61.32)	
Marital status				< 0.01
Married	56.8(54.84–58.74)	57.35(53.41–61.20)	56.62(54.36–58.85)	
Widowed	1.22(0.89–1.68)	1.08(0.54–2.15)	1.27(0.89–1.81)	
Divorced	8.80(7.75–9.98)	10.25(8.05–12.96)	8.32(7.16–9.65)	
Separated	2.85(2.30–3.53)	3.15(2.07–4.75)	2.75(2.14–3.54)	
Never married	19.00(17.55–20.54)	14.52(12.12–17.30)	20.47(18.74–22.32)	
Living with their partners	7.35(6.42–8.41)	9.64(7.50-12.31)	6.60(5.62–7.75)	
Poverty income ratio				< 0.01
<1.3	18.16(16.76–19.65)	24.77(21.63–28.20)	15.98(14.48–17.62)	
1.3–1.8	9.87(8.77–11.08)	10.97(8.75–13.66)	9.50(8.28–10.89)	
>1.8	65.70(63.84–67.51)	57.44(53.52–61.27)	68.42(66.32–70.44)	
Not recorded	6.28(5.41–7.27)	6.82(5.14–9.01)	6.10(5.12–7.25)	
Smoking status				0.15
Low	10.52(9.33–11.85)	11.07(8.76–13.88)	10.35(8.98–11.89)	
Moderate	55.50(53.52–57.46)	52.12(48.15–56.08)	56.61(54.33–58.86)	
High	33.18(31.34–35.07)	35.87(32.14–39.78)	32.29(30.20-34.46)	
Not recorded	0.797(0.52–1.23)	0.94(0.41–2.13)	0.75(0.45–1.24)	
Alcohol consumption				< 0.01
Non drinking	10.48(9.33–11.75)	12.71(10.29–15.60)	9.74(8.47–11.18)	
Moderate drinking	30.14(28.34–32.01)	27.49(24.05–31.22)	31.01(28.92–33.19)	
Heavy drinking	45.63(43.66–47.60)	43.91(40.00-47.89)	46.19(43.92–48.47)	
Not recorded	13.76(12.46–15.13)	15.90(13.32–18.86)	13.06(11.63–14.62)	
Stroking status				< 0.01
Yes	1.09(0.75–1.59)	2.25(1.30–3.86)	0.71(0.42–1.19)	
No	98.86(98.36–99.21)	97.63(96.01–98.60)	99.26(98.79–99.56)	
Not recorded	0.05(0.01–0.20)	0.12(0.02–0.84)	0.03(0.00-0.18)	
Sedentary behavior				< 0.01
Yes	31.69(29.91–33.53)	36.66(32.96–40.53)	30.05(28.04–32.14)	
No	67.20(65.34-69.00)	61.03(57.13–64.79)	69.23(67.12–71.25)	
Not recorded	1.11(0.77–1.60)	2.31(1.38–3.83)	0.72(0.42–1.22)	
Diabetes				0.24
Yes	5.73(4.8–6.75)	6.56(4.86–8.78)	5.45(4.46–6.65)	
No	94.27(93.25–95.15)	93.44(91.22–95.14)	94.55(93.35–95.54)	
Cancer				< 0.01
Yes	4.30(3.54–5.22)	6.40(4.60–8.83)	3.61(2.84–4.58)	
No	95.58(94.65–96.35)	93.23(90.71–95.10)	96.35(95.38–97.13)	
Not recorded	0.12(0.03–0.46)	0.37(0.07–2.03)	0.04(0.01–0.16)	
Body Mass Index (kg/m^2^)	27.93 ± 0.13	28.85 ± 0.28	27.63 ± 0.14	< 0.01
≤ 18.5	1.91(1.41–2.57)	1.80(0.99–3.25)	1.94(1.37–2.74)	
18.5–25	35.35(33.47–37.28)	30.33(26.80–34.10)	37.01(34.81–39.26)	
25–30	33.59(31.76–35.48)	32.67(29.08–36.47)	33.90(31.78–36.08)	
≥ 30	29.15(27.39–30.97)	35.20(31.49–39.10)	27.16(25.19–29.21)	
Fasting insulin (pmol/L)	66.92 ± 1.14	71.87 ± 2.75	65.30 ± 1.214	< 0.01
C-reactive protein	0.40 ± 0.02	0.47 ± 0.04	0.37 ± 0.02	< 0.01
Body wight (kg)	81.15 ± 0.42	81.37 ± 0.90	81.07 ± 0.48	0.72
Height (cm)	170.15 ± 0.20	167.60 ± 0.39	170.96 ± 0.226	< 0.01
Waist circumference(cm)	94.92 ± 0.33	96.02 ± 0.69	94.56 ± 0.367	0.02
LAP	47.55 ± 0.82	51.87 ± 1.81	46.12 ± 0.92	< 0.01
CMI	0.66 ± 0.01	0.69 ± 0.02	0.65 ± 0.01	0.05
VAI	100.96 ± 1.75	2.00 ± 0.06	1.78 ± 0.03	< 0.01
BRI	4.68 ± 0.04	5.00 ± 0.09	4.57 ± 0.05	< 0.01
WTI	6.87 ± 0.01	6.91 ± 0.02	6.86 ± 0.01	0.02
WHtR	0.56 ± 0.00	0.57 ± 0.00	0.55 ± 0.00	< 0.01
TyG	6.86 ± 0.01	6.88 ± 0.02	6.85 ± 0.01	0.14
CI	12.65 ± 0.02	12.69 ± 0.04	12.64 ± 0.02	0.11
WWI	1.20 ± 0.00	1.21 ± 0.01	1.19 ± 0.00	< 0.01

Note: Values are weighted mean ± SE or weighted % (95% confidence interval). P values are weighted

After a rigorous screening process, 3354 participants who were older than 20 years and younger than 60 years were enrolled in this study, of which 839 had severe headache or migraine. The overall crude weighting prevalence of migraine was 24.76% (95% CI: 23.09–26.51), and patients are inclined to be females [65.83% (95% CI: 62.00–69.47) in females vs. 34.17% (95% CI: 30.53–38.01) in males]. Furthermore, those participants with severe headache or migraine tended to lower education levels, more divorced, lower income, fewer alcohol consumption, more sedentary behavior (all *P* < 0.05), and more probable to have elevated CRP levels (0.47 ± 0.04 vs. 0.37 ± 0.02), WC (96.02 ± 0.69 vs. 94.56 ± 0.37), BMI (28.85 ± 0.28 vs. 27.63 ± 0.14), LAP (51.87 ± 1.81 vs. 46.12 ± 0.92), CMI (0.65 ± 0.01 vs. 0.69 ± 0.02), VAI (2.00 ± 0.06 vs. 1.78 ± 0.03), BRI (5.00 ± 0.09 vs. 4.57 ± 0.05), WTI (6.91 ± 0.02 vs. 6.86 ± 0.01), WHtR (0.57 ± 0.00 vs. 0.55 ± 0.00), WWI (1.21 ± 0.01 vs. 1.19 ± 0.00) (all *P* < 0.05), with higher occurrence of mental health problems [3.19% (95% CI: 2.50,4.06) vs. 6.32% (95% CI: 4.71,8.42)], cancer [6.40% (95% CI: 4.60,8.83) vs. 3.61% (95% CI: 2.84,4.58)] (all *P* < 0.05). However, there are no discernible variations in the CI and TyG indices between participants with and without migraine (*P* = 0.12, 0.13, respectively).

### The association of lipid-related obesity metrics with severe headache or migraine

The partial correlation among lipid-related obesity indicators and migraine both in continuous and categorical analyses are illustrated in Table 2. As the continuous analysis demonstrated, positive associations were consistently found between WHtR, BRI, BMI, LAP, WTI, VAI and headache in Models 1–3 (all *P* < 0.05). A strong association between the prevalence of migraines and WWI was observed in Models 1 [odds ratio (OR) = 2.52, 95% CI: 1.31–4.85, *P* < 0.01], however, this association was not stable both in model 2 and model 3 (*P* = 0.26, 0.05, respectively). Furthermore, there were no relationships have been found between TyG, CI, CMI and severe headache or migraine in model1 (all *P* > 0.05). These results finally demonstrated that TyG, CI, CMI cannot perform well in differentiating or forecasting migraine. Among the aforementioned measures, WHtR exhibited the strongest diagnosis ability for migraine (OR = 5.77, 95% CI: 1.93–17.26, *P* < 0.01, in model 3).

**Table 2:** The associations of the quartiles of lipid-related obesity indicators with severe headache or migraine

	Total	Events yes	Model 1	Model 2	Model 3
			OR (95% CI) p-Value	OR (95% CI) p-Value	OR (95% CI) p-Value
WHtR			10.13(3.82,26.86) < 0.01	9.92(3.59,27.38) < 0.01	6.38(2.25,18.09) < 0.01
WHtRQ1	855	215	reference	reference	reference
WHtRQ2	651	218	1.39(1.09,1.78) < 0.01	1.59(1.23,2.05) < 0.01	1.57(1.21,2.03) < 0.01
WHtRQ3	541	209	1.59(1.23,2.05) < 0.01	1.77(1.35,2.32) < 0.01	1.71(1.30,2.25) < 0.01
WHtRQ4	468	197	1.84(1.41,2.40) < 0.01	1.86(1.40,2.47) < 0.01	1.68(1.26,2.25) < 0.01
P for trend			*P* < 0.01	*P* < 0.01	*P* < 0.01
BRI			1.10(1.06,1.15) < 0.01	1.10(1.05,1.15) < 0.01	1.08(1.03,1.13) < 0.01
BRIQ1	855	215	reference	reference	reference
BRIQ2	651	218	1.39(1.09,1.78) < 0.01	1.59(1.23,2.05) < 0.01	1.57(1.21,2.03) < 0.01
BRIQ3	541	209	1.59(1.23,2.05) < 0.01	1.77(1.35,2.32) < 0.01	1.71(1.30,2.25) < 0.01
BRIQ4	468	197	1.84(1.41,2.40) < 0.01	1.86(1.40,2.47) < 0.01	1.68(1.26,2.25) < 0.01
P for trend			*P* < 0.01	*P* < 0.01	*P* < 0.01
BMI			1.03(1.02,1.04) < 0.01	1.03(1.02,1.04) < 0.01	1.03(1.01,1.04) < 0.01
BMIQ1	679	208	reference	reference	reference
BMIQ2	639	172	1.01(0.77,1.31) 0.96	1.15(0.87,1.50) 0.32	1.16(0.88,1.54) 0.28
BMIQ3	604	205	1.12(0.87,1.46) 0.36	1.35(1.03,1.77) 0.03	1.36(1.03,1.79) 0.03
BMIQ4	593	254	1.63(1.27,2.10) < 0.01	1.72(1.33,2.24) < 0.01	1.65(1.26,2.16) < 0.01
P for trend			*P* < 0.01	*P* < 0.01	*P* < 0.01
LAP			1.00(1.00,1.01) < 0.01	1.01(1.00,1.01) < 0.01	1.00(1.00,1.01) 0.02
LAPQ1	841	242	reference	reference	reference
LAPQ2	609	198	1.22(0.95,1.56) 0.13	1.30(1,1.68) 0.05	1.26(0.97,1.65) 0.08
LAPQ3	551	202	1.31(1.02,1.68) 0.04	1.55(1.19,2.02) < 0.01	1.46(1.11,1.91) < 0.01
LAPQ4	514	197	1.46(1.13,1.88) < 0.01	1.70(1.29,2.23) < 0.01	1.54(1.16,2.05) < 0.01
P for trend			*P* < 0.01	*P* < 0.01	*P* < 0.01
WTI			1.16(1.00,1.34) 0.05	1.38(1.17,1.61) < 0.01	1.28(1.09,1.51) < 0.01
WTIQ1	808	255	reference	reference	reference
WTIQ2	616	203	1.06(0.83,1.36) 0.65	1.20(0.93,1.54) 0.17	1.12(0.86,1.46) 0.39
WTIQ3	560	191	1.18(0.92,1.53)0.19	1.46(1.12,1.90) < 0.01	1.34(1.02,1.76) 0.04
WTIQ4	531	190	1.26(0.97,1.62)0.08	1.63(1.24,2.14) < 0.01	1.44(1.09,1.91) 0.01
P for trend			0.05	*P* < 0.01	0.01
VAI			1.11(1.05,1.18) < 0.01	1.11(1.05,1.18) < 0.01	1.07(1.01,1.15) 0.03
VAIQ1	656	183	reference	reference	reference
VAIQ2	637	201	1.27(0.98,1.66) 0.08	1.23(0.94,1.61) 0.13	1.23(0.93,1.61) 0.15
VAIQ3	611	227	1.48(1.14,1.92) < 0.01	1.49(1.13,1.94) < 0.01	1.41(1.07,1.85) 0.02
VAIQ4	611	228	1.50(1.16,1.96) < 0.01	1.54(1.18,2.03) < 0.01	1.36(1.02,1.80) 0.04
P for trend			*P* < 0.01	*P* < 0.01	0.02
WWI			2.52(1.31,4.85) < 0.01	0.65(0.30,1.38) 0.26	0.45(0.21,1.00) 0.05
WWIQ1	661	178	reference	reference	reference
WWIQ2	645	193	1.07(0.82,1.40) 0.60	0.99(0.76,1.30) 0.95	0.99(0.75,1.31) 0.94
WWIQ3	611	227	1.31(1.01,1.71) 0.04	0.97(0.73,1.28) 0.83	0.92(0.69,1.22) 0.55
WWIQ4	598	241	1.34(1.04,1.74) 0.03	0.82(0.61,1.11) 0.20	0.75(0.55,1.02) 0.07
P for trend			0.01	0.21	0.04
TyG			1.11(0.95,1.29) 0.18	1.30(1.11,1.53) < 0.01	1.21(1.02,1.44) 0.03
TyGQ1	821	256	reference	reference	reference
TyGQ2	639	214	1.07(0.84,1.37) 0.57	1.19(0.93,1.54) 0.17	1.13(0.87,1.46) 0.35
TyGQ3	557	191	1.23(0.96,1.59) 0.11	1.48(1.13,1.93) < 0.01	1.36(1.04,1.79) 0.03
TyGQ4	498	178	1.21(0.93,1.57) 0.16	1.56(1.18,2.07) < 0.01	1.39(1.03,1.86) 0.03
P for trend			0.08	*P* < 0.01	0.01
CI			1.07(0.97,1.19) 0.18	1.24(1.10,1.38) < 0.01	1.16(1.03,1.30) 0.02
CIQ1	848	285	reference	reference	reference
CIQ2	754	237	0.97(0.77,1.22)0.77	1.14(0.90,1.45)0.29	1.10(0.86,1.41) 0.45
CIQ3	575	183	0.89(0.69,1.14)0.36	1.15(0.89,1.50)0.29	1.09(0.83,1.43) 0.54
CIQ4	338	134	1.24(0.94,1.65)0.13	1.66(1.22,2.25) < 0.01	1.40(1.01,1.93) 0.04
P for trend			0.42	*P* < 0.01	0.07
CMI			1.15(0.98,1.35)0.08	1.35(1.15,1.58) < 0.01	1.22(1.03,1.45) 0.02
CMIQ1	754	236	reference	reference	reference
CMIQ2	637	198	1.10(0.86,1.41)0.46	1.21(0.94,1.57)0.14	1.19(0.92,1.55) 0.19
CMIQ3	560	211	1.32(1.02,1.69)0.03	1.64(1.26,2.14) < 0.01	1.53(1.17,2.00) < 0.01
CMIQ4	564	194	1.22(0.95,1.58)0.13	1.60(1.22,2.10) < 0.01	1.40(1.05,1.86) 0.02
P for trend			0.05	*P* < 0.01	0.01

Model 1: Non-adjusted model; Model 2 adjusted for: Sex, age, race; Model 3 adjusted for: age, sex, race, education attainment, marital situation, alcohol consumption, smoking status, poverty income ratio, sedentary lifestyle, history of cancer, diabetes, mental health problems

As illustrated by the categorical analysis, in comparison with the first quartile (Q1), which was used as a control group, the significant associations between WHtR, BRI and migraine persisted in the other three quartiles (Q2–Q4). Moreover, with the escalation of WHtR and BRI, the OR values for severe headache or migraine likewise increased (all *P* for trend < 0.01). Parallelly, BMI, LAP, and WTI were intimately tied to escalated headache risk in the fourth quartile (Q4). More precisely, Q4 of WHtR (OR = 1.68, 95% CI: 1.26–2.25, in model 3), BRI (OR = 1.68, 95% CI: 1.26–2.25, in model 3), BMI (OR = 1.65, 95% CI: 1.26–2.16, in model 3), LAP (OR = 1.54, 95% CI: 1.16–2.05, in model 3) and VAI (OR = 1.36, 95% CI: 1.02–1.80, in model 3) exhibited a significant positive relationship with severe headache or migraine in comparison to Q1–3. In conclusion, higher levels of those five obesity-related indicators are independent risk factors for severe headache or migraine.

Intriguingly, while the continuous analysis indicated a positive linkage from WTI to severe headache or migraine, the categorical analysis revealed insignificant association in any of the quartiles in model 1, suggesting the instable associations between WTI and migraine. Besides, in spite of the continuous analysis demonstrated no association between CMI and migraine, CMIQ3 (CMI 0.54–0.94) had a noticeable association with migraine in Models 1–3.

In addition, stratified analysis by age revealed that WHtR, BRI, BMI, LAP, WTI, VAI, TyG, and CI could increase the risk of developing migraine for those aged 20 to 40 (unadjusted model OR = 37.56, 95% CI: 9.85–143.16; OR = 1.17, 95% CI: 1.10–1.24; OR = 1.05, 95% CI: 1.03–1.07; OR = 1.01, 95% CI: 1.00–1.01; OR = 1.30, 95% CI: 1.06–1.59; 1.12, 95% CI: 1.03–1.22; OR = 1.25, 95% CI: 1.01–1.54; OR = 1.18, 95% CI: 1.03–1.37, respectively, all *P* < 0.05). Additionally, positive relationship have been found between WWI and migraine among individuals aged 40 to 60 (unadjusted model OR = 3.00, 95% CI: 1.10–8.15, *P* = 0.03). However, there was no association between CMI and migraine in analyses stratified by age, consistent with the results of the continuous analyses (Supplemental material Table 3).

Following gender stratification, the OR values of the connections between WHtR, BRI, BMI, LAP, WTI, VAI, TyG and CMI could increase the potential for developing severe headache or migraine in females (OR = 4.58, 95% CI: 1.23–7.01; OR = 1.06, 95% CI: 1.00–1.12; OR = 1.02, 95% CI: 1.01–1.04; OR = 1.01, 95% CI: 1.00–1.01; OR = 1.37, 95% CI: 1.10–1.71; OR = 1.10, 95% CI: 1.01–1.19; OR = 1.33, 95% CI: 1.05–1.68; OR = 1.35, 95% CI: 1.05–1.73, in model 3, respectively, all *P* < 0.05) (Supplemental material Table 3).

### Results of stratified analysis and interaction analysis

**Fig. 3:**
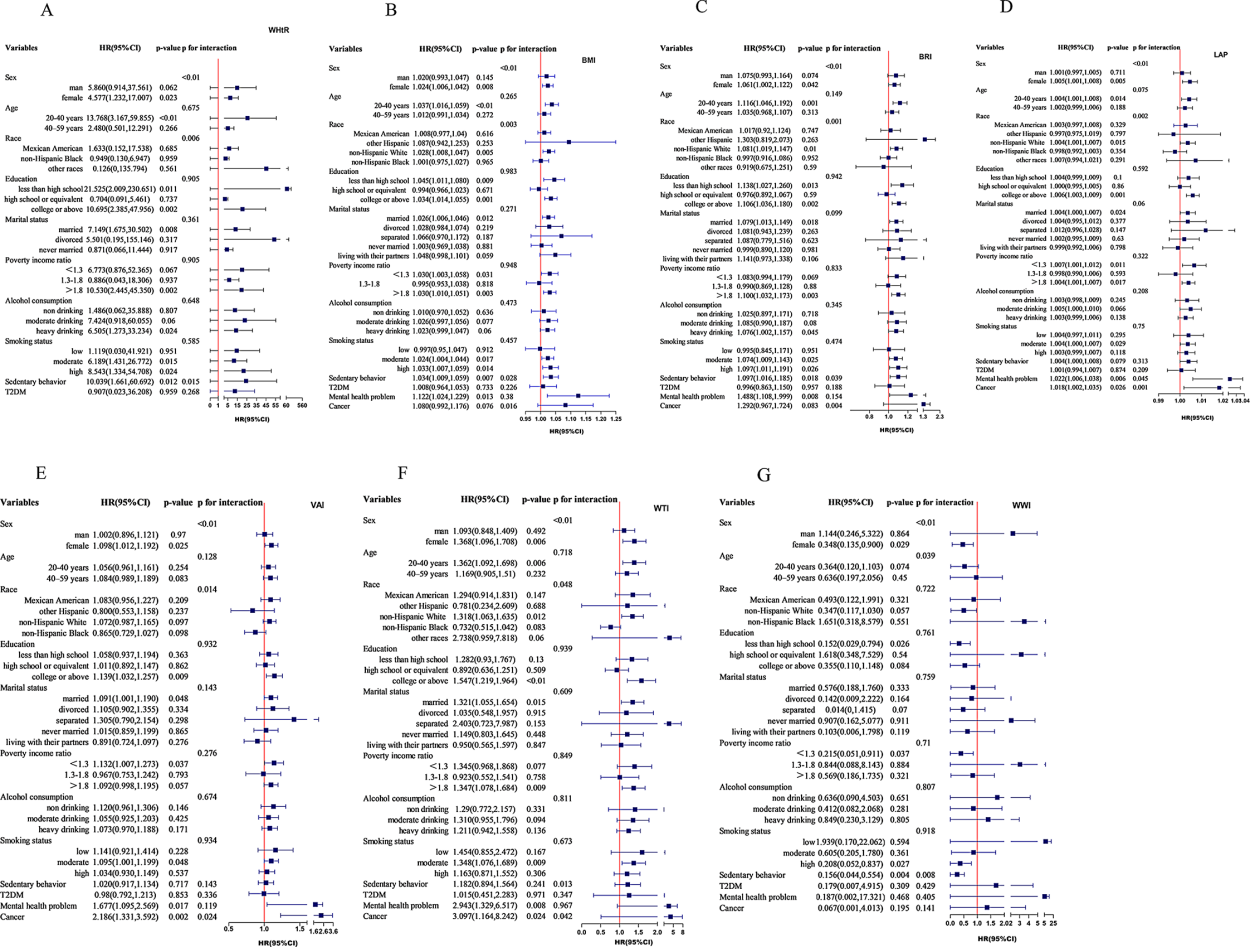
Subgroup and interaction analyses of the association of lipid-related obesity indicators and severe headache or migraine

Stratified analysis and interaction analysis were used to further investigate the role of covariables and lipid-related obesity measures (WHtR, BMI, LAP, WTI, BRI, VAI, WWI) on migraine (Fig. 3).

In the stratified analysis, which was stratified by race, WHtR, BMI, BRI, WTI, LAP were substantially associated with migraine among non-Hispanic White people (OR: 6.73, 95% CI: 1.70–26.69; OR: 1.03, 95% CI: 1.01–1.05; OR: 1.08, 95% CI: 1.02–1.15; OR: 1.32, 95% CI: 1.06–1.64; OR: 1.00, 95% CI: 1.00–1.01, respectively). Statistically significant associations were observed between WHtR, BMI, VAI, BRI, LAP and WTI with severe headache or migraine in individuals who were married, higher educational level, higher income. Additional examination of interactions showed that WHtR, BMI, BRI and WTI significantly influenced the risk of migraines in relation to age, race, sedentary behavior and history of cancer (*P* for interaction < 0.05). Additionally, significant interactions were identified between VAI, LAP and age, race, history of cancer (*P* for interaction < 0.05). These results could partly explain why WWI were not any more relevant with migraine in Model 2–3.

### Nonlinear associations between lipid-related obesity metrics and the prevalence of migraine

**Fig. 4:**
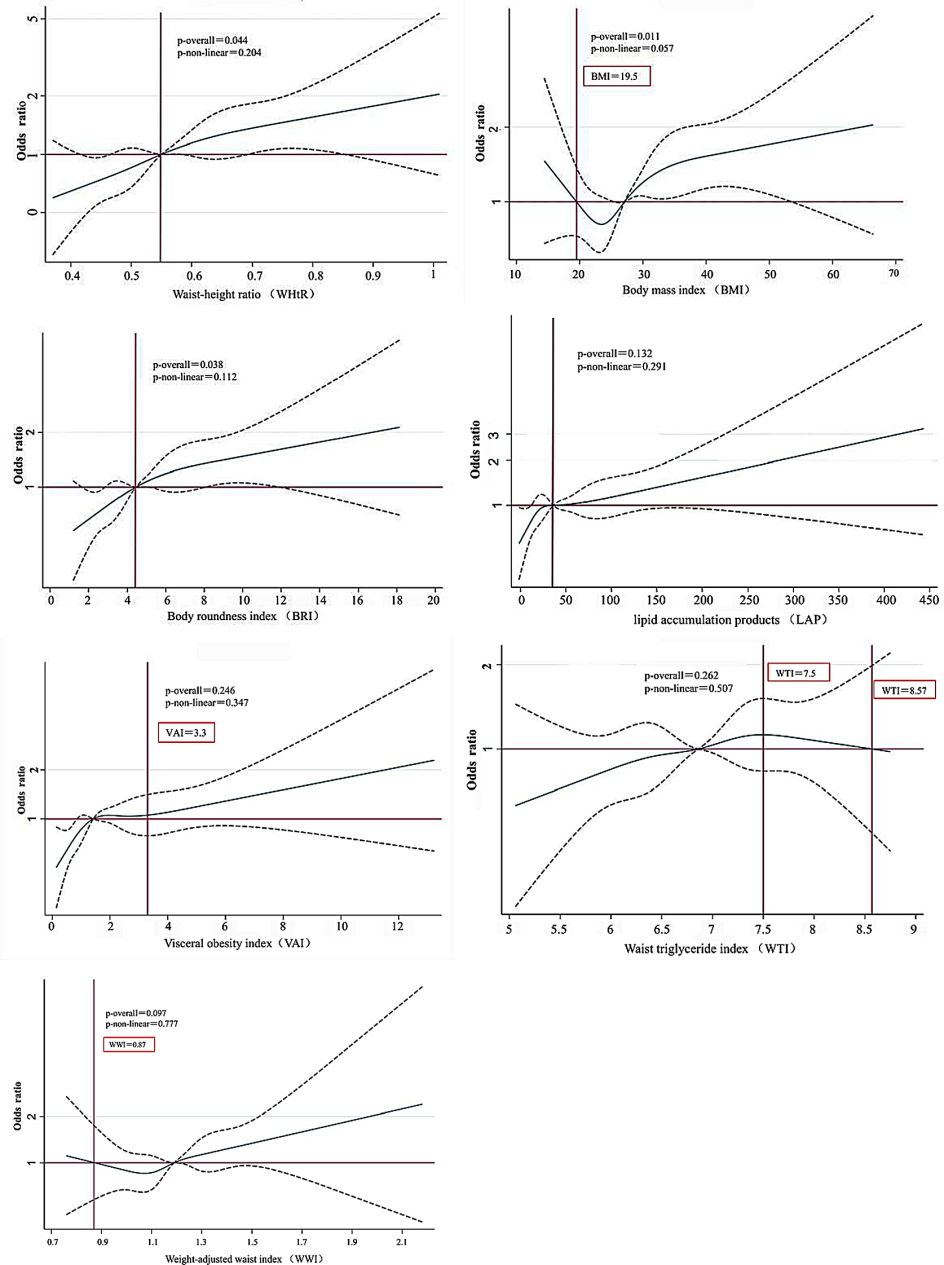
Restricted cubic splines of lipid-related obesity indicators and severe headache or migraine. Note: The dashed lines represent the 95% confidence interval; the red lines represent inflection points.

In combination with the positive outcomes of the logistic regression analyses, we employed RCS to visually demonstrate dose–response relationships the associations between WHtR, BMI, LAP, WTI, BRI, VAI, WWI and the prevalence of migraine in model1, and the median value of those seven indicators (WHtR = 0.56, BMI = 27.14, LAP = 36.20, WTI = 6.86, BRI = 4.44, VAI = 1.42, WWI = 1.19) were used as the reference point (Fig. 4).

Among those seven indicators, WHtR, BMI and BRI demonstrated dose-response relationships with the prevalence of migraine (*P* for overall = 0.04, 0.01, 0.04, respectively). However, dose-response relationships between LAP, WTI, VAI, WWI and migraine are not significant (*P* for overall = 0.13, 0.26, 0.25, 0.10, respectively). Furthermore, the RCS plot demonstrated linear relationships between WHtR, BMI and BRI and the risk of migraine (*P* for non-linearity = 0.20, 0.06, 0.11, respectively), more precisely, the likelihood of experiencing severe headache or migraine rises as BMI, BRI, and WHtR increase.

### ROC curves of lipid-related obesity indicators in relation to severe headache or migraine

**Fig. 5:**
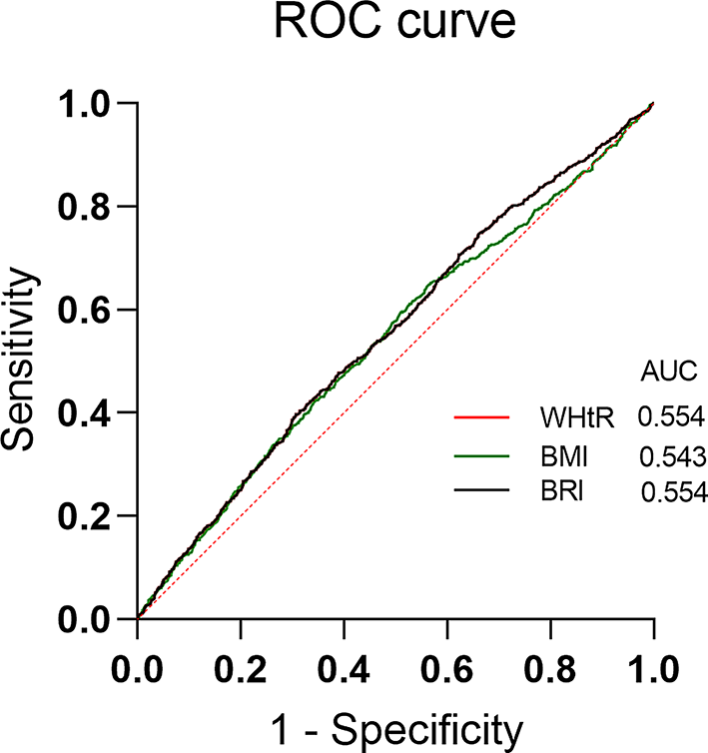
Receiver operating characteristic curves of WHtR, BMI, BRI and severe headache or migraine

The ROC curve indicated that WHtR and BRI had comparable and highest diagnostic efficacy for severe headache or migraine (AUC: 0.55, 95% CI: 0.53–0.58; AUC: 0.55, 95% CI: 0.53–0.58), slightly higher specificity compared to BMI (AUC: 0.54, 95% CI: 0.52–0.57) (Fig. 5).

## Discussion

Throughout this cross-sectional investigation, we inaugurally investigated the association between ten lipid-related obesity indices and migraine in young and middle-aged Americans, and observed that WHtR, BRI, BMI, LAP, WTI and VAI were all associated with migraine. Amongst the above indicators evaluated, WHtR showed the best diagnostic accuracy in predicting migraine risk and is less influenced by confounding factors, which supports previous clinical and epidemiological studies that WHtR has a higher sensitivity and association than BMI in discriminating migraine risk. Moreover, WWI were connected to overall migraine, but the connections were not stable after adjusting for confounding factors. The unstable results could partly be explained by the data derived from our interaction analysis that the significant interaction of sex, age and sedentary behavior with WWI on migraine.

A wealth of data supports that using more sensitive anthropometric measurements for different populations could improve the accuracy of predicting their vulnerability to migraine, which was demonstrated by the subgroup analysis we performed on several anthropometric measures based on age and sex [25]. Our gender-stratified analysis revealed that WHtR, BRI, BMI, LAP, WTI, VAI, TyG and CMI were more effective in predicting migraine risk in women. Consistent with our findings, several studies have confirmed that lipid-related obesity indices are more sensitive in predicting the risk of migraine in women [26, 27]. Sex differences in the results may be attributed to the sex hormonal fluctuations, human overall adipose tissue composition exhibits sexual dimorphism [28]. Specifically, adipose tissue in females is often subcutaneously stored, particularly gluteo-femoral distribution, whereas men tend to accumulate more abdominal adipose tissue depots [29].

In the age-stratified analysis on reproductive-age men and women, the majority of anthropometric characteristics (WHtR, BRI, BMI, LAP, WTI, VAI, TyG, and CI) were observed to be more successful in estimating the risk of migraine for those aged 20 to 40. The finding indicated a tendency towards a more robust association among younger age cohorts, which was consistent with previous research [12]. The underlying mechanism for these results may be associated with changes in the hormone levels of these individuals around childbearing age. Noteworthy, considering that ageing is related to changes in fat distribution, and anthropometric measures have been demonstrated to lack specificity in detecting obesity-related risk in older adults, we excluded the data of those older than 60. As Peterlin BL’s study revealed that association with migraine was significantly attenuated in older adults with obesity [30].

As illustrated by the categorical analysis, higher levels of WHtR, BRI, BMI, LAP and WTI are considered independent risk factors for migraine. Considering interaction and hierarchical models, there remained a robust positive association between obesity and migraine. Furthermore, the RCS indicated that WHtR, BMI and BRI demonstrated linear dose-response relationships with the prevalence of migraine (all *P*_overall_ < 0.05, *P*_non−linearity_ > 0.05). Our finding implies that there might be complexity in their relationship, and higher levels of those three indicators are related with considerably increased prevalence of migraine. The ROC results showed that whereas WHtR, BMI, and BRI were substantially linked with migraine, they had limited clinical diagnostic value. The low AUC may be due to the instability and overfitting of the variables resulting in insufficient model generalization ability. In addition, the definition of migraine in this study was based on the NHANES database questionnaire, although most self-reported severe headaches met the ICHD-II criteria for migraine, which may be insufficiently rigorous. These two reasons may be contribute to the limited predictive value of a single migraine headache.

Anthropometric data is frequently employed as surrogates for overall or abdominal adiposity to assess disease risk, since it is a clearly comprehensible, conveniently available, and low-cost tool for epidemiological surveys [31, 32]. Recent studies have evaluated the bidirectional links between migraine and obesity, since certain pathogenic determinants are similar between the two situations. The BRI was suggested by Thomas et al. [33], as a trustworthy indicator of body fat percentage and visceral adiposity, which has been proven show efficacy in depicting the intricate relationship between lipid toxicity and Mets [34]. The BMI is extensively utilized in ordinary research due to its simplicity in height and weight calculations. Nevertheless, rather than focusing on abdominal obesity, most previous studies have primarily considered the effects of BMI, a general obesity index derived from self-reported weight and height, on migraine attacks characteristics [35]. The WHtR has gained increasing attention in relation to abdominal obesity, as it is closely associated with abdominal fat measured, also can quantify visceral fat distribution [36]. Furthermore, relevant literature has previously conducted comparative analyses between WHtR and BMI in the evaluation of the danger associated with obesity and indicated that WHtR has demonstrated superior sensitivity to BMI in predicting relative metabolic diseases, including diabetes mellitus, cardiovascular diseases [37, 38]. Our finding that the higher sensitivity and closer association of WHtR than BMI in discriminating migraine risk is in alignment with former studies. An Isfahan research found a substantial association between WC, WHtR with the regularity and intensity of migraine episodes [39]. However, the study conducted by Santos et al. in the Brazilian Longitudinal Study of Adult Health (ELSA-Brasil) revealed that there was no discernible association between Abd-O and BMI with migraine, which exhibit certain disparities compared to our results [40]. Differences may be due to the fact that the ELSA-Brasil study is conducted on a sample of urban civil servants that may not be representative of the entire population. In addition, Kristoffersen et al. [41] suggested that TBO may be a better indicator of obesity than Abd-O in identifying migraine risk. The following two factors may be the primary cause of the discrepancy: (1) Kristoffersen et al. used the third Nord-Trondelag Health Study (HUNT3) database from Norway, a big sample database targeted at long-term track the change tendency of diseases. (2) The age of the study population was different, Kristoffersen et al. screened the population between 19 and 96 years old, while the older population has been proven there is less of abdominal fat. In our opinion, above disparities may primarily be attributed to variations in sample composition and the regional disparities in the study population.

The association between migraine and obesity was initially investigated in a patient-based research, which discovered that obese individuals had a threefold higher risk of migraine than those age-matched normal weight controls [42]. However, studies examining the correlation between lipid-related obesity markers and migraine have yielded conflicting results. For example, this research not found any noteworthy independent association between TyG, CMI, CI and migraine in young and middle-aged patients. However, Liu et al. [43] demonstrated a linear relationship result between TyG and migraine. Variations in the study’s results may primarily stem from the fact that Liu’s study was screening the NHANES database from 1999 to 2018 and that the diagnostic criteria for inclusion of migraine patients varied. Zhuang et al. [44] showed that elevated levels of LAP and VAI were associated with higher migraine risk in U.S. adults, which was generally consistent with the trend of the results in the present study, although the results were slightly different due to the inconsistent inclusion of the population. However, this study intended to comprehensively evaluate the diagnostic significance of ten lipid-related obesity indicators for severe headache or migraine, and to compare the diagnostic utility of these measures with more thorough and objective analysis. Differences amongst the study’s results could primarily stem from the effects of genetic polymorphisms, physical activity, culture, nutrition, and ethnicity on migraine attack characteristics, and also the extraction of data from various inclusion and exclusion criteria [45, 46].

The underlying pathophysiological mechanisms of obesity driving the migraine remain elusive, involving overlapping pathways implicated in the etiology of headache as well as cerebral and peripheral pathways that influence the function of adipose tissue [47]. Hypothalamus plays an essential role in connecting obesity and migraine processes, the hypothalamus’s neural circuitry is a key brain area involved in the pathogenesis of headaches, and the arcuate nucleus (ARC) is necessary for managing appetite and food intake [48–50]. Besides, various neurotransmitters and neuropeptides synthesized by the hypothalamus could possibly be crucial molecular mediators connecting obesity and headache. Compared to subcutaneous fat, visceral adipose tissue (VAT) primarily adheres to abdominal organs, contains more inflammatory and immune cells, more vascular and nerve supply, and a higher metabolic activity [51]. The proinflammatory state of VAT is thought to accelerate cardiovascular and multiple metabolic diseases in obese patients [52]. Furthermore, both migraine and obesity are chronic low-grade inflammatory and pro-thrombotic states, such as central sensitization caused by chronic serotonin deficiency, hyperleptinemia, and elev/ated plasma levels of interleukin-6 (IL-6), tumour necrosis factor-a (TNF-a), CRP, P-molecule and calcitonin gene-related peptide (CGRP) [53–58]. Specifically, dysregulation of lipid metabolism leads to increased levels of inflammatory mediators, including interleukin and calpain gene-related peptide (CGRP), causing vasodilation, mast cell degranulation and plasma extravasation, which in turn leads to migraine attacks [59].

### Strengths and limitations

The present research demonstrates several significant advantages. Firstly, this study is the first to comprehensively assess the correlation between lipid-related indices and migraine in young and middle-aged adults. Since anthropometric indices may have reduced predictive accuracy in older adults, we restricted our analyses to young and middle-aged people can eliminate the interference of some confounding factors, so as to make the results more convincing. Secondly, considering that previous studies have primarily considered the effect of a single indicator of obesity on migraine attacks, we comprehensively and synthetically evaluated 10 lipid-related measures and performed comparative analyses yielding more sensitive and specific anthropometric measures to improve diagnosis ability in specific migraine patients. Thirdly, the NHANES database, on which our analysis is based, is a comprehensive survey that makes use of strict quality control processes, established study methodologies, and maximize the elimination of potential sources of measurement bias for physiological and biochemical indicators. Fourthly, DAG provides a graphical and systematic methodology to minimizing confounding bias and avoiding inadequate adjustment or overadjustment bias in the regression models constructed [60, 61]. Nevertheless, it’s critical to recognize some of our study’s shortcomings. Firstly, anthropometric measurements and migraine cannot be causally or temporally linked according to the cross-sectional methodology employed in this study. Secondly, there still exist some potential confounding variables, including medication use, sleeping patterns, psychological and nutritional status, which may interfere with these associations. Thirdly, migraine status was self-reported, which made it impossible to investigate other parameters, including the frequency, intensity, duration, or subtypes of migraine attack.

## Conclusion

In conclusion, among those ten lipid-related obesity indicators evaluated in our study, WHtR, BMI and BRI demonstrated linear positive dose-response relationships with the prevalence of migraine and WHtR showed the best predictive ability. Our study can provide important insight into epidemiological research and comprehensive management of obese patients with migraine in young and middle-aged individuals, and suggested that weight management might be a helpful migraine treatment tactic. Additionally, large-scale longitudinal studies and robust causal inference will also be pivotal in verifying the current conclusions and establishing a more thorough understanding of the mechanisms behind this link.

## Electronic supplementary material

Below is the link to the electronic supplementary material.

Supplementary Material 1

## Data Availability

No datasets were generated or analysed during the current study.

## Abbreviations

Mets: Metabolic syndrome
Abd-O: Abdominal obesity
TBO: Total body obesity
AMPP: The American migraine prevalence and prevention
CVD: Cardiovascular disease
IR: Insulin resistance
VAT: Visceral adipose tissue
PIR: Poverty income ratio
HbA1c: Glycated Hemoglobin A1c
CRP: C-reactive protein
WT: Body wight
HT: Height
WC: Waist circumference
HDL: High density lipoprotein cholesterol
LAP: Lipid accumulation products
CMI: Cardiometabolic index
VAI: Visceral obesity index
BRI: Body roundness index
WTI: Waist triglyceride index
WHTR: Waist-height ratio
TyG: Triglyceride-glucose
CI: Cone index
WWI: Weight-adjusted waist index
NHANES: National Health and Nutrition Examination Survey
IL-6: Interleukin-6
TNF-a: Tumour necrosis factor-a
CGRP: Calcitonin gene-related peptide
ARC: Arcuate nucleus
RCS: Restricted cubic splines
ROC: Receiver operating characteristic
ELSA-Brasil: The Brazilian Longitudinal Study of Adult Health
ICHD-II: The international classification of headache disorders, 2rd edition
ICHD-3: The international classification of headache disorders, 3rd edition
