# Supplementary material for: The association between lipid-related obesity indicators and severe headache or migraine: a nationwide cross sectional study from NHANES 1999 to 2004
Source: Lipids Health Dis. 2025 Jan 11;24:10. doi: 10.1186/s12944-025-02432-w (PMC11724612; doi:10.1186/s12944-025-02432-w)
Supplement: Supplementary file 1 — Supplementary Material 1 [file 12944_2025_2432_MOESM1_ESM.docx]

Supplementary Material

# Supplementary Table 3

Table3 Associations between lipid-related obesity indicators and severe headache or migraine in analyses stratified by age and gender

| Variables |  | male(n=1,686) | female(n=1,668) | <40 years(n=1,715) | 40–59 years(n=1,639) |
| --- | --- | --- | --- | --- | --- |
|  |  | OR (95% CI) p-Value | OR (95% CI) p-Value | OR (95% CI) p-Value | OR (96% CI) p-Value |
| WHtR |  |  |  |  |  |
|  | Model1 | 10.50(1.94,56.94)<0.01 | 7.75(2.37,25.38)<0.01 | 37.56(9.85,143.16)<0.01 | 2.83(0.62,12.93)0.18 |
|  | Model2 | 10.38(1.73,62.33)0.01 | 8.01(2.26,28.39)<0.01 | 25.63(6.51,100.93)<0.01 | 3.06(0.67,13.89)0.15 |
|  | Model3 | 5.86(0.91,37.56)0.06 | 4.58(1.23,17.01)0.02 | 13.77(3.17,59.86)<0.01 | 2.48(0.50,12.29)0.27 |
| BRI |  |  |  |  |  |
|  | Model1 | 1.11(1.03,1.19)<0.01 | 1.09(1.03,1.14)<0.01 | 1.17(1.10,1.24)<0.01 | 1.05(0.98,1.12)0.17 |
|  | Model2 | 1.11(1.02,1.19)＜0.01 | 1.09(1.03,1.15)<0.01 | 1.15(1.08,1.22)<0.01 | 1.05(0.98,1.12)0.16 |
|  | Model3 | 1.08(0.99,1.16)0.07 | 1.06(1.00,1.12)0.04 | 1.12(1.05,1.19)<0.01 | 1.04(0.97,1.11)0.31 |
| BMI |  |  |  |  |  |
|  | Model1 | 1.03(1,1.05)0.05 | 1.03(1.01,1.05)<0.01 | 1.05(1.03,1.07)<0.01 | 1.01(0.99,1.03)0.29 |
|  | Model2 | 1.03(1.00,1.05)0.06 | 1.03(1.01,1.05)<0.01 | 1.04(1.02,1.07)<0.01 | 1.01(0.99,1.03)0.23 |
|  | Model3 | 1.02(0.99,1.05)0.15 | 1.02(1.01,1.04)0.01 | 1.04(1.02,1.06)<0.01 | 1.01(0.99,1.03)0.27 |
| LAP |  |  |  |  |  |
|  | Model1 | 1.00(1.00,1.01)0.20 | 1.01(1.00,1.01)<0.01 | 1.01(1.00,1.01)<0.01 | 1.00(1.00,1.01)0.32 |
|  | Model2 | 1.00(1.00,1.01)0.31 | 1.01(1.00,1.01)<0.01 | 1.01(1.00,1.01)<0.01 | 1.00(1.00,1.01)0.10 |
|  | Model3 | 1.00(1.00,1.00)0.71 | 1.01(1.00,1.01)0.01 | 1.00(1.00,1.01)0.01 | 1.00(1.00,1.01)0.19 |
| WTI |  |  |  |  |  |
|  | Model1 | 1.21(0.97,1.51)0.10 | 1.42(1.16,1.74)<0.01 | 1.30(1.06,1.59)0.01 | 1.04(0.83,1.31)0.74 |
|  | Model2 | 1.19(0.94,1.51)0.16 | 1.50(1.21,1.85)<0.01 | 1.53(1.24,1.88)<0.01 | 1.18(0.93,1.50)0.17 |
|  | Model3 | 1.09(0.85,1.41)0.49 | 1.37(1.10,1.71)0.01 | 1.36(1.09,1.70)0.01 | 1.17(0.91,1.51)0.23 |
| VAI |  |  |  |  |  |
|  | Model1 | 1.05(0.95,1.16)0.32 | 1.14(1.05,1.23)<0.01 | 1.12(1.03,1.22)<0.01 | 1.10(1.01,1.20)0.02 |
|  | Model2 | 1.04(0.94,1.16)0.41 | 1.15(1.06,1.24)<0.01 | 1.12(1.02,1.22)0.01 | 1.10(1.01,1.20)0.02 |
|  | Model3 | 1.00(0.90,1.12)0.97 | 1.10(1.01,1.19)0.03 | 1.06(0.96,1.16)0.25 | 1.08(0.99,1.19)0.08 |
| WWI |  |  |  |  |  |
|  | Model1 | 1.82(0.50,6.61)0.37 | 0.52(0.22,1.24)0.14 | 2.19(0.93,5.15)0.07 | 3.00(1.10,8.15)0.03 |
|  | Model2 | 1.76(0.43,7.22)0.43 | 0.43(0.17,1.07)0.07 | 0.40(0.14,1.12)0.08 | 0.96(0.31,2.96)0.94 |
|  | Model3 | 1.14(0.25,5.32)0.86 | 0.35(0.14,0.9)0.03 | 0.36(0.12,1.10)0.07 | 0.64(0.20,2.06)0.45 |
| TyG |  |  |  |  |  |
|  | Model1 | 1.12(0.89,1.39)0.34 | 1.37(1.12,1.69)<0.01 | 1.25(1.01,1.54)0.04 | 1.00(0.80,1.25)0.99 |
|  | Model2 | 1.09(0.86,1.39)0.46 | 1.46(1.17,1.82)<0.01 | 1.48(1.19,1.85)<0.01 | 1.11(0.88,1.40)0.37 |
|  | Model3 | 1.03(0.79,1.34)0.83 | 1.33(1.05,1.68)0.02 | 1.30(1.03,1.66)0.03 | 1.11(0.85,1.43)0.45 |
| CI |  |  |  |  |  |
|  | Model1 | 1.29(1.09,1.52)<0.01 | 1.15(1.01,1.32)0.04 | 1.18(1.03,1.37)0.02 | 0.98(0.83,1.16)0.83 |
|  | Model2 | 1.31(1.08,1.59)<0.01 | 1.16(1.00,1.34)0.04 | 1.29(1.11,1.50)<0.01 | 1.14(0.96,1.35)0.13 |
|  | Model3 | 1.21(1.00,1.48)0.06 | 1.07(0.92,1.25)0.38 | 1.19(1.01,1.40)0.03 | 1.11(0.92,1.34)0.30 |
| CMI |  |  |  |  |  |
|  | Model1 | 1.15(0.92,1.44)0.22 | 1.52(1.21,1.91)<0.01 | 1.21(0.97,1.50)0.09 | 1.11(0.88,1.40)0.39 |
|  | Model2 | 1.13(0.90,1.43)0.29 | 1.55(1.23,1.95)<0.01 | 1.40(1.12,1.76)<0.01 | 1.27(1.01,1.59)0.04 |
|  | Model3 | 1.02(0.79,1.32)0.86 | 1.35(1.05,1.73)0.02 | 1.22(0.95,1.56)0.12 | 1.20(0.94,1.54)0.15 |

Model 1: Non-adjusted model; Model 2 adjusted for sex, age, race; Model 3 adjusted for age, sex, race, education attainment, marital situation, alcohol consumption, smoking status, poverty income ratio, sedentary lifestyle, history of cancer, diabetes, mental health problems.

Abbreviation: LAP, lipid accumulation products; CMI, Cardiometabolic index; VAI, visceral obesity index; BRI, body roundness index; WTI, waist triglyceride index; WHtR, waist-height ratio; TyG, triglyceride-glucose; CI, cone index; WWI, weight-adjusted waist index.
